# Predictive value of tumor-infiltrating lymphocytes to pathological complete response in neoadjuvant treated triple-negative breast cancers

**DOI:** 10.1186/s13000-018-0743-7

**Published:** 2018-08-31

**Authors:** Miao Ruan, Tian Tian, Jia Rao, Xiaoli Xu, Baohua Yu, Wentao Yang, Ruohong Shui

**Affiliations:** 1 Department of Pathology, Fudan University Shanghai Cancer Center, Shanghai, China; 2 Department of Oncology, Shanghai Medical College, Fudan University, Shanghai, China

**Keywords:** Tumor-infiltrating lymphocytes, Breast cancer, Triple-negative, Pathological complete response, Predictive factor

## Abstract

**Background:**

Triple-negative breast cancers (TNBCs) are a group of heterogeneous diseases with various morphology, prognosis, and treatment response. Therefore, it is important to identify valuable biomarkers to predict the therapeutic response and prognosis for TNBCs. Tumor-infiltrating lymphocytes (TILs) may have predictive value to pathological complete response (pCR) in neoadjuvant treated TNBCs. However, absence of standardized methodologies for TILs measurement has limited its evaluation and application in practice. In 2014, the International TILs Working Group formulated the recommendations of pathologic evaluation for TILs in breast cancers.

**Methods:**

To evaluate the predictive value of TILs scored by methods recommended by International TILs Working Group 2014, we performed a retrospective study of TILs in 166 core needle biopsy specimens of primary invasive TNBCs with neoadjuvant chemotherapy (NAC) in a Chinese population. Intratumoral TILs (iTILs) and stromal TILs (sTILs) were scored respectively. The associations between TILs and pCR were analyzed.

**Results:**

Both sTILs (*p* = 0.0001) and iTILs (*P* = 0.001) were associated with pCR in univariate logistic regression analysis. Multivariate logistic regression analysis indicated that both sTILs (*P* = 0.006) and iTILs (*P* = 0.04) were independent predictors for pCR. Receiver operating characteristics (ROC) curve analysis was used to identify the optimal thresholds of TILs. TNBCs with more than 20% sTILs (*P* = 0.001) or with more than 10% iTILs (*P* = 0.003) were associated with higher pCR rates in univariate analysis. Multivariate analysis showed that a 20% threshold of sTILs (*P* = 0.005) was an independent predictive factor for pCR.

**Conclusions:**

Our study indicated that TILs scored by recommendations of International TILs Working Group 2014 in pre-NAC core needle biopsy specimens was significantly correlated with pCR in TNBCs, higher TILs scores predicting higher pCR rate. Both sTILs and iTILs were independent predictors for pCR in TNBCs. A 20% threshold for sTILs may be feasible to predict pCR to NAC in TNBCs.

## Background

Triple-negative breast cancers (TNBCs) are defined as a group of breast cancer characterized by lacking of estrogen receptor (ER), progesterone receptor (PgR) and human epidermal growth factor receptor 2 (HER2) protein expression [1]. Most of TNBCs have higher risk of early distant recurrence, mortality and more aggressive clinical behavior compared with other subtypes of breast cancers [2, 3]. Owing to the absence of effective targeted therapy, chemotherapy is the only recommended systemic treatment for TNBCs at present stage [4]. However, various therapeutic strategies have been explored, among which immunotherapy may have potential benefits to treat TNBCs [5, 6]. Therefore, several studies have been carried out to evaluate the predictive and prognostic values of tumor-infiltrating lymphocytes (TILs) in TNBCs, which have indicated that high levels of TILs may be associated with a better clinical outcome and a better response to chemotherapy in TNBCs [7–9].

It has been demonstrated that patients gaining pathological complete response (pCR) to neoadjuvant chemotherapy (NAC) may experience prolonged disease-free survival, especially in TNBCs [3, 7, 8]. Many biomarkers to predict pCR for NAC in TNBCs have been analyzed, such as immune-related gene signatures and clinicopathologic factors. Several studies have indicated that TILs in pre-NAC samples may be used to predict pCR in TNBCs [9–12]. However, methodologies of TILs evaluation in these studies were not standardized, which has hindered its application in clinical practice.

Lymphocyte-predominant breast cancer (LPBC), firstly proposed by Denkert et al., was defined as tumors with a particularly strong lymphocytic infiltration whether in tumor stroma or cell nests [9]. The LPBC-cutoff was generally 50% or 60% in previous literatures [9, 13]. However, owing to the relatively low proportion of LPBC in routine practice, it may be unreasonable to define 50–60% as the threshold for LPBC.

In this study, we conducted a retrospective analysis of TILs in 166 core needle biopsy specimens of TNBCs with NAC in a Chinese population. Stromal TILs (sTILs) and intratumoral TILs (iTILs) in pre-NAC specimens were scored using the method recommended by the International TILs Working Group 2014 [14], and the correlation between TILs and neoadjuvant chemotherapy response was analyzed. The optimal thresholds of TILs to predict pCR in TNBCs were explored. The aim of our study was to examine the predictive value of TILs to pCR in neoadjuvant treated TNBCs, and to evaluate the feasibility of the scoring methods in clinical practice.

## Methods

### Patients and samples

166 consecutive core needle biopsy specimens of primary invasive TNBCs diagnosed and treated with NAC following up operation between 2011 and 2016 were extracted from the pathology database of Fudan University Shanghai Cancer Center. The inclusion criteria were as follows: primary invasive TNBCs; neoadjuvant therapy before surgical operation; available complete clinicopathologic data (age, tumor size, tumor grade, histological type, lymphovascular invasion, lymph node status, Miller-Payne grade, ER, PgR, HER2 and Ki-67 index). All specimens were fixed with 10% neutral phosphate-buffered formalin and paraffin-embedded. 4 μm-thick slices of representative tumor blocks were stained with hematoxylin and eosin (H& E). Tumors were defined as triple negative as following: < 1% of ER and PgR immunoreactivity, and absence of HER2 protein overexpression or gene amplification [15, 16].

### Pathologic evaluation

All core needle biopsy specimens and surgical slices were reviewed by two experienced breast pathologists (R.S. and W.Y.) to confirm the histological type, according to 2012 World Health Organization (WHO) Classification of Tumours of the Breast [17]. Histological grade of tumor was evaluated in pre-NAC core needle biopsy specimens by the Nottingham grading system [18, 19]. Miller-Payne grading system was used to evaluate the pathological response in surgical specimens [20]. The pCR of chemotherapeutic response was the endpoint of our study, which was defined as the absence of invasive carcinoma in the breast tissue and axillary lymph nodes in surgical specimens.

**Fig. 1:**
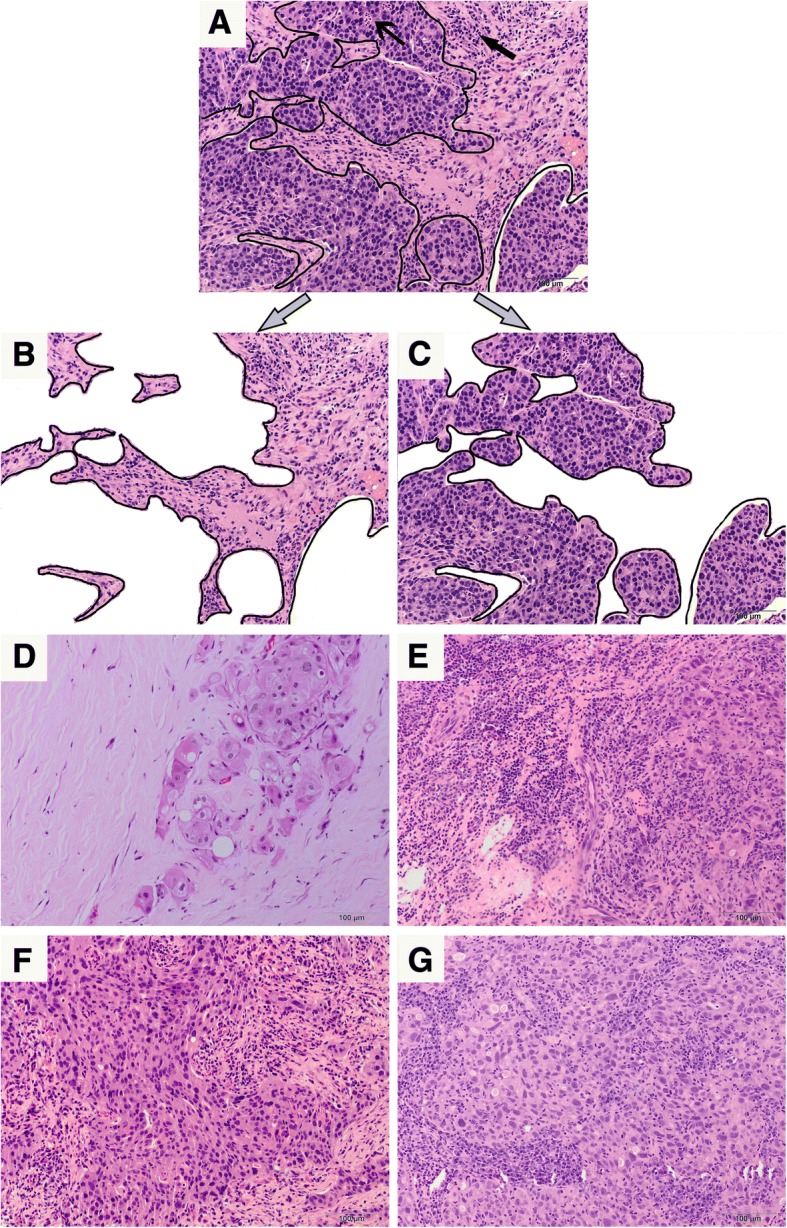
Histopathologic evaluation of TILs and different scores of TILs in TNBCs. **a**: The areas of sTILs and iTILs evaluation were distinguished by black lines (bold black arrow pointed to plasma cells and lymphocytes, Fine black arrow pointed to polymorphonuclear leukocytes which should be excluded in TILs evaluation). **b**: The area of sTILs evaluation: 20%; **c**: The area of iTILs evaluation: 0%. **d**: sTILs: 0%. **e**: sTILs: 50%. **f**: iTILs: 10%. **g**: iTILs: 20% (All × 200 magnification)

Evaluation of TILs on core needle biopsy specimens was performed by two breast pathologists (M.R. and T.T.). The two observers were trained by the evaluation criteria recommended by the International TILs Working Group 2014, and scored each case independently in a blind manner. The mean values of two observers were obtained as final scores for each case. STILs were defined as the percentage of tumor stroma containing infiltrating lymphocytes and plasma cells, which should exclude polymorphonuclear leukocytes (Fig. 1a, b). ITILs were defined as the percentage of lymphocytes and plasma cells within tumor cell nests or in direct contact with the tumor cells (Fig. 1a, c). Areas of in situ carcinomas, normal lobules, necrosis, hyalinization and crush artifacts were not included [14]. The TILs were scored in an average value throughout full sections rather than hotspots. The results were scored in increments of 10; 0 was defined as < 5%; 10 was defined as 5% to 10%; 20 was defined as 11% to 20% and all other scores were rounded up to the next highest decile (Fig. 1d-1g).

### Statistical analysis

Two types of variables were used to test: one was continuous variables (per 10% increment); the other was binary variables categorized by 20% score cutoff (sTILs) and 10% score cutoff (iTILs). The associations between TILs and clinicopathologic characteristics including patients’ age (≤ 50 years versus > 50 years), tumor size (≤ 2 cm, 2–5 cm versus > 5 cm), tumor grade (1, 2 versus 3), lymph node status (negative versus positive), histological type (IDC versus special type), LVI (negative versus positive), Ki-67 index, Miller-Payne grade (1–5) and neoadjuvant chemotherapy regiments were analyzed. The correlations of TILs as continuous variables with polytomous variables were evaluated with Kruskal-Wallis test. Mann-Whitney test was performed to identify the associations between TILs and binary variables. The associations between TILs and continuous variables (Ki-67 index and Miller-Payne grade) were evaluated with Spearman’s rank correlation analysis (r). The intraclass correlation coefficient analysis was used to evaluate the interobserver agreement of sTILs and iTILs scores. The correlation between pCR after NAC and TILs was analyzed by univariate logistic regression analysis. Multivariate logistic regression analysis was used to identify the independent predictors for chemotherapeutic response. Stratified analysis was used to investigate the correlation between TILs and pCR across different clinicopathologic subgroups. Evaluation of heterogeneity effects and test for trend were performed by chi-squared test. Receiver operating characteristics (ROC) curve was conducted to detect the optimal thresholds of TILs and the predictive model to predict pCR. The maximum Youden’s Index (*J* = sensitivity + specificity - 1) was calculated to define the optimal thresholds, then univariate and multivariate regression analysis were used to evaluate the predictive value of TILs as binary variables for pCR. A two-side *p-*value < 0.05 was considered statistically significant. All statistical analyses were performed using the SPSS version 20.0 (SPSS Inc., Chicago, IL) and STATA version 13.1 (Stata Corporation, College Station, TX, USA).

## Results

### Clinicopathologic characteristics

The clinicopathologic characteristics of 166 TNBCs were listed in Table 1. The age of patients ranged from 25 to 77 years with a mean age of 50 years. All patients were female. The pre-NAC tumor size was assessed according to radiology findings. The tumor grade was estimated in the pre-NAC specimens of core needle biopsy. In core needle biopsy, 164 (98.8%) cases were diagnosed as invasive ductal carcinoma of no special type (IDC) and only 2 (1.2%) cases were invasive carcinoma with special subtypes (carcinoma with apocrine differentiation in 2 cases). Lymph nodes involvement was found in 113 (68.1%) cases by fine needle aspiration (FNA) before neoadjuvant treatment. Patients underwent NAC based on the combination treatment regimen of anthracycline and paclitaxel (136/166, 81.9%), or paclitaxel and platinum (30/166, 18.1%). All patients received surgical operation after eight cycles of NAC. 141 (84.9%) patients underwent mastectomy, and breast-conserving surgery was performed in 25 (15.1%) patients. Lymphovascular invasion (LVI) was observed in the postoperative slices of 36 (21.7%) cases. 67 (40.4%) cases obtained pCR, and non-pCR were observed in 99 (59.6%) cases.

**Table 1:** Correlations between TILs and clinicopathologic characteristics in TNBCs

Characteristics	No. of patients (%)	*P*-value of sTILs	*P*-value of iTILs
Age (years)			
< 50	80 (48.2)	0.87^a^	0.79^a^
≥ 50	86 (51.8)		
Tumor size (cm)			
≤ 2	22 (13.2)	0.88^b^	0.16^b^
2–5	115 (69.3)		
> 5	29 (17.5)		
Lymph node status			
Negative	53 (31.9)	0.78^a^	0.23^a^
Positive	113 (68.1)		
Histological type			
IDC	164 (98.8)	0.58^a^	0.33^a^
Special type	2 (1.2)		
Histological grade			
2	31 (18.7)	0.004^a+^	0.03^a+^
3	135 (81.3)		
LVI			
Negative	130 (78.3)	0.17^a^	0.03^a+^
Positive	36 (21.7)		
Miller-Payne grade			
1	17 (10.2)	0.001^b+^	0.06^b^
2	26 (15.7)		
3	32 (19.3)		
4	24 (14.4)		
5	67 (40.4)		
NAC			
Anthracycline + paclitaxel	136 (81.9)	0.98^a^	0.99^a^
Paclitaxel + platinum	30 (18.1)		

Abbreviations: *TNBCs* triple-negative breast cancers, *sTILs* stromal tumor-infiltrating lymphocytes, *iTILs* intratumoral tumor-infiltrating lymphocytes, *IDC* invasive ductal carcinoma of no special type, *LVI* lymphovascular invasion, *NAC* neoadjuvant chemotherapy

^a^Mann-Whitney test

^**b**^Kruskal-Wallis test

^**+**^The *p* value is significant

### Correlations between TILs and clinicopathologic parameters

The distribution of TILs in 166 core needle biopsy specimens was summarized in Table 2. The average score of sTILs was 15% (range: 0–60%), and the average score of iTILs was 5% (range: 0–30%). STILs score was positively associated with iTILs (*r* = 0.65, *p* < 0.001) by Spearman correlation analysis. Using the intraclass correlation coefficient (ICC) analysis, the interobserver agreement of sTILs and iTILs assessment both were excellent (sTILs: ICC 0.91, 95% CI 0.88–0.93, *P* = 0.001; iTILs: ICC 0.83, 95% CI 0.77–0.88, *P* = 0.001).

**Table 2:** The distribution of TILs scores in TNBCs

Score (%)	Cancer with sTILs No. (%)	Cancer with iTILs No. (%)
0	17 (10.2)	95 (57.2)
10	80 (48.2)	64 (38.6)
20	41 (24.7)	5 (3)
30	21 (12.7)	2 (1.2)
40	0	0
50	4 (2.4)	0
60	3 (1.8)	0
70	0	0
80	0	0
90	0	0
100	0	0

Abbreviations: *TNBCs* triple-negative breast cancers, *sTILs* stromal tumor-infiltrating lymphocytes, *iTILs* intratumoral tumor-infiltrating lymphocytes

**Fig. 2:**
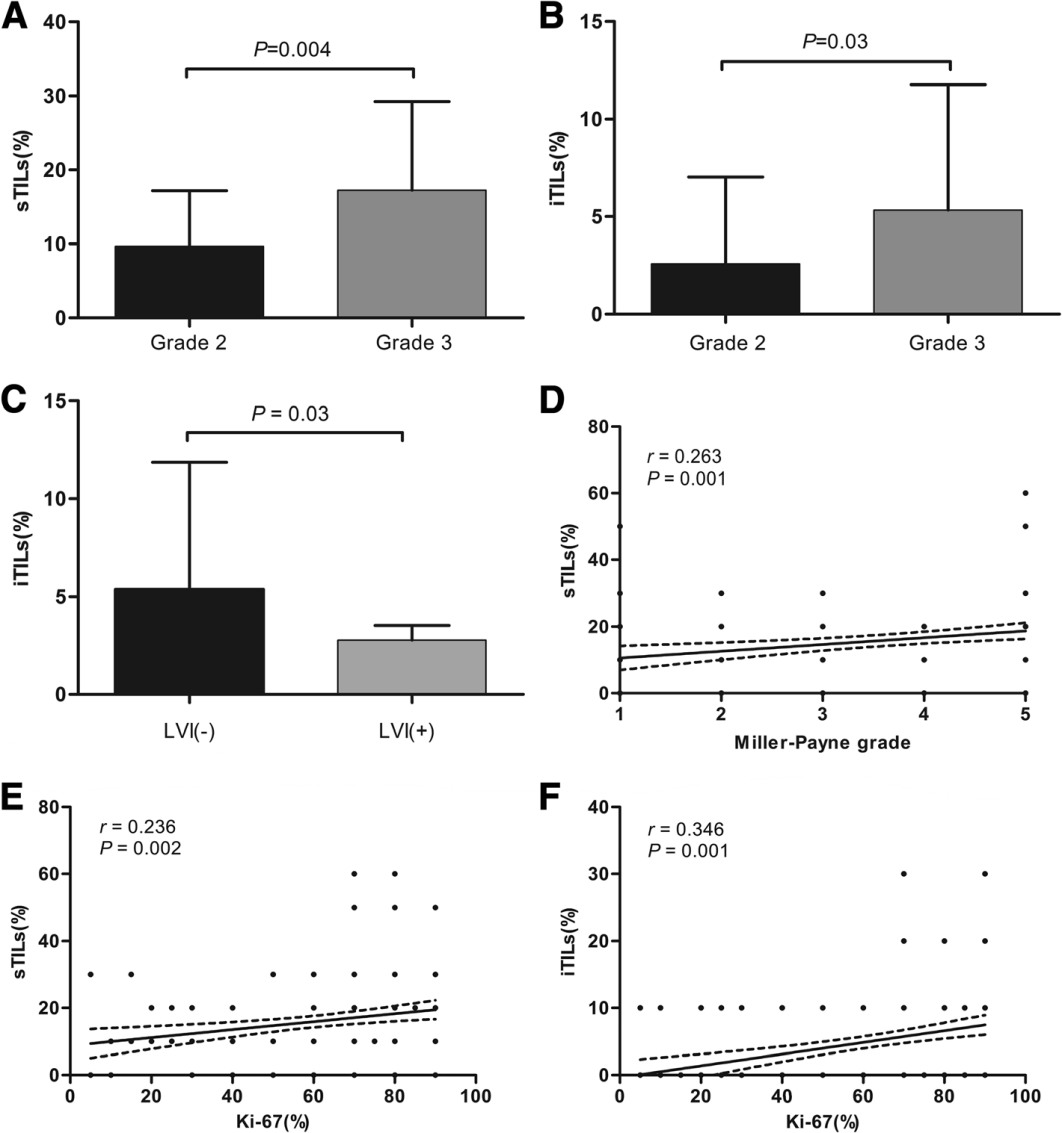
The correlations of TILs with histological grade, LVI, Miller-Payne grade and Ki-67 index in TNBCs. Y axis represented the scores of TILs; X axis represented histological grade (2 or 3), LVI (−/+), Miller-Payne grade (1–5) or Ki-67 index (%). **a** and **b**: Both sTILs and iTILs were positively associated with histological grade (sTILs: *P* = 0.004; iTILs: *P* = 0.03). **c**: ITILs were positively correlated with negative lymphovascular invasion (*P* = 0.03). Dots corresponded to the scores of TILs and whiskers corresponded to its Standard Error. Red line corresponded to the mean scores of TILs. **d:** STILs was positively correlated with Miller-Payne grade (*r* = 0.263, *P* = 0.001). **e** and **f**: STILs and iTILs scores were positively associated with Ki-67 index (sTILs: *r* = 0.236, *P* = 0.002; iTILs: *r* = 0.346, *P* = 0.001). LVI: lymphovascular invasion

The correlations between clinicopathologic characteristics and TILs were analyzed in Table 1. Mann-Whitney test showed that both sTILs (*P* = 0.004) and iTILs (*P* = 0.03) were positively associated with histological grade (Fig. 2a, b), tumors with grade 3 having more lymphocytic infiltration than grade 2. ITILs were positively correlated with negative lymphovascular invasion (*P* = 0.03, Fig. 2c). Spearman’s rank correlation analysis revealed that sTILs were positively correlated with Miller-Payne grade, higher sTILs scores in pre-NAC specimens having higher Miller-Payne grade after operation (*r* = 0.263, *P* = 0.001, Fig. 2d). Spearman’s rank correlation analysis showed that both higher scores of sTILs (*r* = 0.236, *P* = 0.002) and iTILs (*r* = 0.346, *P* = 0.001) were positively related with higher Ki-67 index in TNBCs (Fig. 2e, f). There was no significant association of TILs with patients’ age, tumor size, lymph node status, histological type and neoadjuvant chemotherapy regimens.

### Correlations between TILs and pCR

The relationship between TILs and pCR was analyzed by logistic regression analysis (Table 3). TILs were scored as continuous variables (per 10% increment). Univariate analysis showed that both sTILs (per 10% sTILs: OR 1.07, 95% CI 1.03–1.10, *P =* 0.0001) and iTILs (per 10% iTILs: OR 1.10, 95% CI 1.04–1.16, *P* = 0.001) were significantly correlated with pCR. Higher TILs scores indicated higher pCR rate. Multivariate analysis demonstrated that both sTILs (per 10% sTILs: OR 1.05, 95% CI 1.02–1.09, *P* = 0.006) and iTILs (per 10% iTILs: OR 1.06, 95% CI 1.00–1.12, *P* = 0.04) were independent predictors for pCR, irrespective of other clinicopathologic factors.

**Table 3:** Correlations between TILs and pCR in neoadjuvant treated TNBCs

Variables	Univariate analysis			Multivariate analysis		
	OR	95% CI	*P-*value	OR	95% CI	*P*-value
ITILs (per 10%)	1.10	1.04–1.16	0.001*	1.06	1.00-1.12	0.04*
STILs (per 10%)	1.07	1.03–1.10	0.0001*	1.05	1.02-1.09	0.006*
Age (years) (< 50 vs. ≥50)	0.51	0.27–0.95	0.04*	0.65	0.31-1.35	0.25
Histological grade (2 vs. 3)	2.74	1.11–6.80	0.03*	1.02	0.34-3.08	0.98
Tumor size (cm) (≤2 vs. 2–5 vs. > 5)	1.30	0.74–2.29	0.37	1.30	0.68–2.47	0.43
Nodal status (negative vs. positive)	0.93	0.48–1.81	0.84	1.52	0.72–3.22	0.28
LVI (negative vs. positive)	0.06	0.01–0.26	0.0001*	0.07	0.02-0.29	0.0001*
Ki-67 index	1.03	1.01–1.04	0.001*	1.02	1.006-1.04	0.009*
NAC (Anthracycline + paclitaxel vs. paclitaxel + platinum)	0.83	0.37–1.87	0.65	1.10	0.41–2.94	0.85

Abbreviations: *pCR* pathological complete response, *TNBCs* triple-negative breast cancers, *sTILs* stromal tumor-infiltrating lymphocytes, *iTILs* intratumoral tumor-infiltrating lymphocytes, *LVI* lymphovascular invasion, *NAC* neoadjuvant chemotherapy, *OR* odds ratio, *95% CI* 95% confidence interval

*The *P* value is significant

Stratified analysis was used to investigate whether the predictive value of TILs might be different in every subgroup of clinicopathologic characteristics (Table 4). The chi-squared test showed that there was no significant difference for TILs predicting the rate of pCR in each subgroup (*p* > 0.05).

**Table 4:** Correlations between TILs and pCR in clinicopathologic subgroups

Variable	No. of patients	Per 10% sTILs increase			Per 10% iTILs increase		
		Adjusted OR	95% CI	*P*-value*	Adjusted OR	95% CI	*P*-value*
Age (years)							
< 50	80	1.06	1.01–1.11	0.78	1.09	1.01–1.18	0.64
≥ 50	86	1.07	1.02–1.12		1.12	1.03–1.22	
Tumor size (cm)							
≤ 2	22	1.05	0.99–1.12	0.68	1.07	0.92–1.25	0.72
2–5	115	1.06	1.02–1.10		1.09	1.02–1.16	
> 5	29	1.11	1.00–1.24		1.16	1.00–1.35	
Lymph node status							
Negative	53	1.07	1.00–1.14	0.81	1.07	0.96–1.18	0.56
Positive	113	1.06	1.02–1.10		1.11	1.04–1.19	
Grade							
2	31	1.01	0.90–1.13	0.34	1.02	0.84–1.23	0.46
3	135	1.07	1.03–1.10		1.11	1.04–1.17	
LVI							
Negative	130	1.06	1.02–1.10	0.49	1.08	1.02–1.15	0.84
Positive	36	1.14	0.93–1.39		1.11	0.83–1.48	
NAC							
Anthracycline + paclitaxel	136	1.07	1.03–1.12	0.23	1.12	1.06–1.20	0.14
Paclitaxel + platinum	30	1.01	0.93–1.10		1.00	0.87–1.14	

Abbreviations: *pCR* pathological complete response, *sTILs* stromal tumor-infiltrating lymphocytes, *iTILs* intratumoral tumor-infiltrating lymphocytes, *LVI* lymphovascular invasion, *NAC* neoadjuvant chemotherapy, *OR* odds ratio, *95% CI* 95% confidence interval

*The chi-squared test

### The optimal thresholds of TILs to predict pCR

**Fig. 3:**
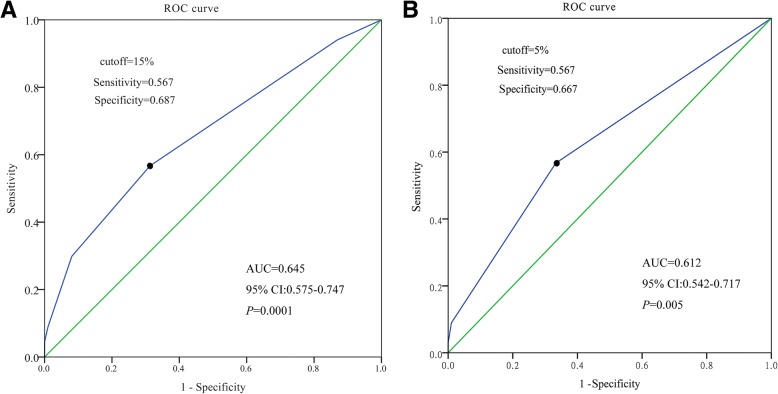
Receiver operating characteristics (ROC) analysis for the thresholds of TILs to predict pCR in neoadjuvant treated TNBCs. ROC curves of sTILs **(a)** and iTILs **(b)**. The black dot indicated the optimal threshold. The area under the curve (AUC), 95% confidence interval (CI) and *P*-value were listed in the picture

Receiver operating characteristics (ROC) curve analysis was used to identify the optimal thresholds of TILs distinguishing pCR from non-pCR cases (Fig. 3). It was revealed that the area under the curve (AUC) of sTILs level was 0.645 (95% CI 0.575–0.747, *P* = 0.0001) and the best cutoff value of sTILs to predict pCR was 15%. The AUC of iTILs level was 0.612 (95% CI 0.542–0.717, *P* = 0.005) and the best cutoff value of iTILs was 5%. Because TILs was scored in 10% increments in our study, all cases were categorized into two groups respectively according to the results of ROC curve: TNBCs with sTILs ≥20% and TNBCs with sTILs < 20%; TNBCs with iTILs ≥10% and TNBCs with iTILs < 10%. The sensitivity, specificity, positive predictive value (PPV), negative predictive value (NPV), accuracy and Youden’s Index of the 20% threshold for sTILs and the 10% for iTILs to predict pCR were showed in Table 5.

**Table 5:** Comparisons between different thresholds of sTILs and iTILs

Threshold (%)	No. of patients (%)	Sensitivity (%)	Specificity (%)	PPV (%)^a^	NPV (%)	Accuracy (%)	Youden’s Index
STILs							
20	41.6	56.7	68.7	55.1	70.1	63.9	0.254
50	4.2	9.0	99.0	85.7	61.6	62.7	0.08
60	1.8	4.5	100.0	100.0	60.7	61.4	0.045
ITILs							
10	42.8	56.7	66.7	53.5	69.5	62.7	0.234
20	4.2	8.9	98.9	85.7	61.6	62.7	0.078
30	1.2	2.9	100.0	100.0	60.4	60.8	0.029

Abbreviations: *sTILs* stromal tumor-infiltrating lymphocytes, *iTILs* intratumoral tumor-infiltrating lymphocytes, *PPV* positive predictive value, *NPV* negative predictive value, Youden’s Index = sensitivity + specificity - 1

^a^PPV = pCR rate

Meanwhile, 20% threshold of sTILs was compared with 50% and 60% thresholds. The sensitivity, specificity, PPV, NPV, accuracy and Youden’s Index of these thresholds for sTILs were summarized in Table 5. It was shown that 41.6% of patients had a level of sTILs more than 20%, and the sensitivity and specificity were higher than other thresholds. 10% threshold of iTILs was also compared with 20% and 30% thresholds (Table 5). 42.8% of patients had a level of iTILs more than 10%, and the sensitivity and specificity were higher than other thresholds. Therefore, our study indicated that 20% threshold of sTILs and 10% threshold of iTILs may be optimal to predict pCR in TNBCs.

### Predictive value of the optimal thresholds of TILs

Logistic regression analysis was performed to evaluate the predictive value of the optimal thresholds in our study. As shown in Table 6, TNBCs with more than 20% sTILs (OR 2.87, 95% CI 1.51–5.47, *P* = 0.001) or more than 10% iTILs (OR 2.62, 95% CI 1.38–4.97, *P* = 0.003) both were significantly associated with higher pCR rate in univariate analysis. Multivariate analysis confirmed that a 20% threshold of sTILs (OR 2.85, 95% CI 1.38–5.90, *P* = 0.005) was an independent predictive factor for pCR, while a 10% threshold of iTILs wasn’t an independent predictive factor for pCR (*P* > 0.05).

**Table 6:** Correlations between the optimal thresholds of TILs and pCR in neoadjuvant treated TNBCs

Variables	Univariate analysis			Multivariate analysis		
	OR	95% CI	*P-*value	OR	95% CI	*P*-value
ITILs (< 10% vs. ≥10%)	2.62	1.38–4.97	0.003*	1.97	0.98-3.98	0.06
STILs (< 20% vs. ≥20%)	2.87	1.51–5.47	0.001*	2.85	1.38-5.90	0.005*
Age(years) (< 50 vs. ≥50)	0.51	0.27–0.95	0.04*	0.70	0.33-1.45	0.33
Histological grade (2 vs. 3)	2.74	1.11–6.80	0.03*	1.02	0.34-3.10	0.97
Tumor size (cm) (≤2 vs. 2–5 vs. > 5)	1.30	0.74–2.29	0.37	1.12	0.58–2.14	0.74
Nodal status (positive vs. negative)	0.93	0.48–1.81	0.84	1.76	0.82–3.79	0.15
LVI (positive vs. negative)	0.06	0.01–0.26	0.0001*	0.05	0.01-0.25	0.0001*
Ki-67 index	2.47	1.17–5.24	0.02*	1.03	1.01-1.04	0.004*
NAC (Anthracycline + paclitaxel vs. paclitaxel + platinum)	0.83	0.37–1.87	0.65	0.99	0.49–2.00	0.97

Abbreviations: *pCR* pathological complete response, *TNBCs* triple-negative breast cancers, *sTILs* stromal tumor-infiltrating lymphocytes, *iTILs* intratumoral tumor-infiltrating lymphocytes, *LVI* lymphovascular invasion, *NAC* neoadjuvant chemotherapy, *OR* odds ratio, *95% CI* 95% confidence interval

*The *P* value is significant

**Fig. 4:**
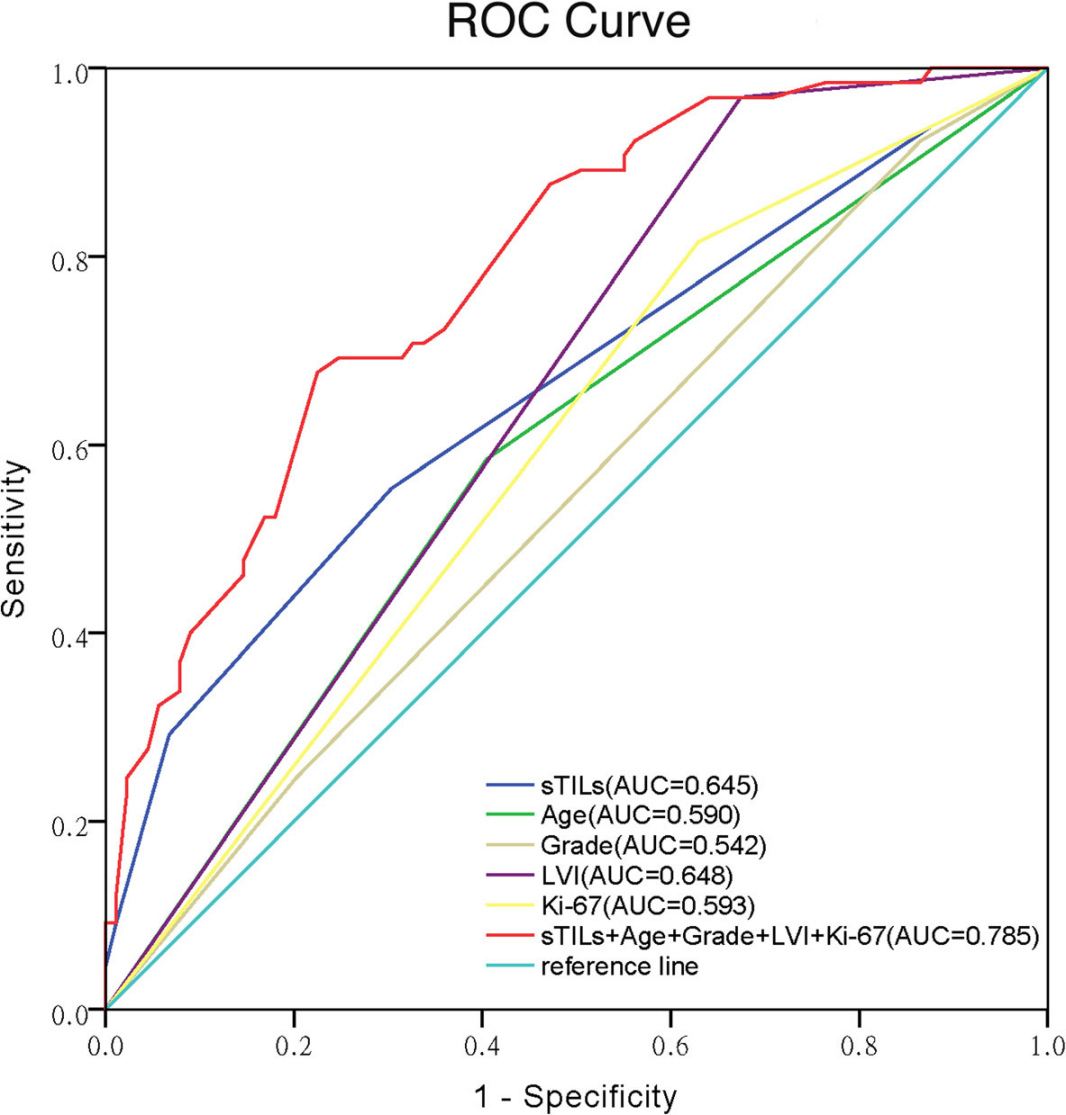
Receiver operating characteristics (ROC) analysis for the combination of sTILs with clinicopathologic parameters (patients’ age, histological grade, LVI and Ki-67 index) to predict pCR in TNBCs. The AUC (area under the curve) were listed in the picture. 95% confidence interval (CI) of combined predictive model was 0.714–0.856 (*P* = 0.0001), sTILs: 95% CI 0.575–0.747 (*P* = 0.0001), patients’ age: 95% CI 0.499–0.681(*P* = 0.057), histological grade: 95% CI 0.450–0.634 (*P* = 0.373), LVI: 95% CI 0.562–0.733 (*P* = 0.002), Ki-67: 95% CI 0.503–0.683 (*P* = 0.049)

In view of the relatively low sensitivity and specificity of the 20% threshold for sTILs to independently predict pCR (56.7% and 68.7%, respectively), a predictive model for response to NAC was performed by combining sTILs with clinicopathologic parameters which had a significant association with the rate of pCR in univariate analysis (including patients’ age, histological grade, LVI and Ki-67 index) (Table 6). ROC curve analysis demonstrated that the AUC for the combination of these five variables was 0.785 (95% CI 0.714–0.856, *P* = 0.0001), and the sensitivity and specificity to predict pCR were 77.6% and 72.7%, respectively. And it’s indicated that the combined predictive model may be more optimal to predict pCR than these clinicopathologic parameters alone in TNBCs (Fig. 4).

## Discussion

TNBCs are a group of heterogeneous diseases with various morphology, prognosis, and treatment response. Therefore, it is important to identify valuable biomarkers to predict the therapeutic response and prognosis for TNBCs. Several studies have demonstrated the prognosis and the predictive values of TILs in TNBCs [12, 21–24]. However, absence of standardized methodologies for TILs measurement has limited its evaluation and application in practice. In 2014, the International TILs Working Group formulated the recommendations of pathologic evaluation for TILs in breast cancers. The recommendations need to be further validated in multiple laboratories before application in routine practice. The study of Pruneri et al. supported the validity of TILs evaluation recommendations in the clinical practice, indicating that each 10% increase in TILs strongly predicted better survival in TNBCs [25]. However, Park et al*’*s study showed that TILs scored by the recommendations may not be useful for predicting survival outcomes in early TNBCs [26]. In our previous study, we carried out a retrospective analysis of TILs in 425 primary invasive TNBCs using the recommendations, indicating that TILs scored by the recommendations could be associated with the prognosis of TNBCs [27]. In this study, we performed a retrospective analysis of TILs in 166 core needle biopsy specimens of TNBCs with NAC, aimed to evaluate the predictive value of TILs scored by the recommendations to pCR. Our study indicated that TILs scored by the recommendations in pre-NAC core needle biopsy of TNBCs were significantly correlated with pCR, higher TILs score strongly predicting higher pCR rate, and TILs score was an independent predictor for pCR. Another issue which should be evaluated is the reproducibility of the recommendations. Swisher et al. evaluated the interobserver agreement of TILs scored by recommendations among four observers, and showed an acceptable agreement in TILs evaluation [28]. In our study, an excellent interobserver agreement between two observers was demonstrated in TILs evaluation. However, large-scale investigation should be performed to assess the intra- and inter-observer reproducibility of TILs evaluation before the application of TILs assessment in clinical practice.

Several studies have evaluated the predictive value of sTILs and iTILs for pCR in neoadjuvant treated TNBCs [12, 21, 29–32]. Khoury et al. found that both sTILs and iTILs were independent predictors for pCR in TNBCs [33]. Denkert et al’s study revealed that iTILs was a significant independent parameter for pCR in breast cancers in both training and validation cohorts, while sTILs was a strong predictor for pCR just in validation cohort [9]. The study of Issa-Nummer et al. showed that, in HER2-negative breast cancer, sTILs was a significant independent predictor for pCR in multivariate analysis, while iTILs was significant for pCR only in univariate but not in multivariate analysis [34]. Our study indicated that both higher sTILs and iTILs score strongly predicted higher pCR rate in univariate analysis, and both sTILs and iTILs score were independent predictors for pCR in multivariate analysis in TNBCs.

The cutoff value of TILs to predict therapeutic response has been analyzed in previous studies. Some studies found that lymphocyte-predominant breast cancer (LPBC, defined as involving ≥50% or ≥ 60% lymphocytic infiltration of either tumor stroma or cell nests) was an independent predictor of pCR for neoadjuvant treated triple-negative and HER2-positive breast cancers [22, 34–36]. However, the 2014 International TILs Working Group recommendations suggested that it was arbitrary to define 50–60% as the threshold for LPBC, because of the relatively low proportion of these cases in breast cancers [14]. In our previous study, only 3.5% of TNBCs had more than 50% lymphocytes [27]. In this study, only 7 of 166 TNBCs (4.2%) had more than 50% lymphocyte infiltration in core needle biopsy specimens. It was unsuitable to define a cutoff value of 50% as LPBC to predict pCR because of the limited clinical implication caused by the low proportion of these cases. In our study, ROC curve analysis revealed that 20% threshold of sTILs and 10% threshold of iTILs may be more optimal to predict pCR compared with other cutoff values. TNBCs with more than 20% sTILs or with more than 10% iTILs were associated with higher pCR rate in univariate analysis. A 20% threshold of sTILs was an independent predictor for pCR in multivariate analysis. However, a 10% threshold of iTILs wasn’t an independent predictor for pCR in multivariate analysis, which may due to the relatively low score of iTILs in our study. In addition, our study performed a predictive model for therapeutic response by the combination of sTILs score, patients’ age, histological grade, LVI and Ki-67 index in TNBCs. It was shown that the combined five variables had relatively high sensitivity and specificity. Therefore, the predictive model might have potential value to predict NAC treated response in TNBCs in practice.

The relationships between clinicopathologic parameters and TILs were also analyzed in our study. It was revealed that both higher scores of sTILs and iTILs were related with higher Ki-67 index and higher histological grade, and higher iTILs scores were positively correlated with negative LVI in TNBCs. Krishnamurti et al. found that TILs was significantly associated with histologic grade 3 in TNBCs [37]. The study of Chung et al. showed that infiltration of CD4+, CD8+, and FOXP3+ TILs was significantly higher in tumors with high Ki-67 index [38]. Pan et al.’s study revealed that the percentage of sTILs and density of CD8+ T-lymphocytes were positively correlated with Ki-67 in TNBCs [39]. Lee et al. found that TNBCs with higher levels of TILs showed lower LVI [40]. However, the relationships between clinicopathologic characteristics and TILs still need to be further explored.

The relationship between TILs subpopulations and therapeutic response has been studied in breast cancers in recent years. Garcia-Martinez et al. and Castaneda et al. found that higher ratio of CD8+/CD4+ was associated with higher pCR rate in pre-NAC breast cancers [11, 12]. The study of Seo et al. revealed that CD8+ TILs was an independent predictor for pCR irrespective of breast cancer subtypes [41]. The research of Asano et al. showed that the pCR rate was significantly higher in the high CD8+/FOXP3+ TIL ratio (CFR) group, and high-CFR status was an independent predictor of a favorable prognosis for TNBC and HER2+ breast cancer [42]. However, the scoring methods of subgroup evaluation were not standardized in these studies, the clinical application of TILs subpopulations still needs more available evidence.

## Conclusions

In summary, our study indicated that TILs scored by methods recommended by International TILs Working Group 2014 in pre-NAC core needle biopsy specimens was significantly correlated with pCR in TNBCs, higher TILs scores predicting higher pCR rate. Both sTILs and iTILs were independent predictors for pCR in TNBCs. A 20% threshold for sTILs may be feasible to predict pCR to NAC in TNBCs. The combination of sTILs and other clinicopathologic variables might have potential value to predict NAC treated response in TNBCs in practice.

## Abbreviations

ER: estrogen receptor
HER2: human epidermal growth factor receptor 2
LPBC: lymphocyte-predominant breast cancer
pCR: pathological complete response
PgR: progesterone receptor
TILs: tumor-infiltrating lymphocytes
TNBCs: triple-negative breast cancers
